# Pulmonary Thromboembolism: A Rare Vascular Complication of Amebic Liver Abscess

**DOI:** 10.7759/cureus.10872

**Published:** 2020-10-10

**Authors:** Gautam Jesrani, Samiksha Gupta, Monica Gupta, Saurabh Gaba, Vivek Naveen

**Affiliations:** 1 General Medicine, Government Medical College and Hospital, Chandigarh, IND

**Keywords:** thrombosis, inferior vena cava, pulmonary thromboembolism, liver abscess, complications, amebic liver abscess

## Abstract

Amebic liver abscess (ALA) is the most common extra-intestinal manifestation of amebiasis. Its complications include rupture into peritoneum, pleural space or anterior abdominal wall. Large abscesses can cause compression of neighboring vascular structures with thrombus formation. Herein, we are presenting an unusual case of a 26-year-old male patient who presented with fever, abdominal pain, chest pain and dyspnea. Ultrasound of the abdomen revealed a lesion in the right lobe of liver and chest radiograph revealed minimal right-sided pleural effusion. A computed tomographic (CT) scan was done in view of unexplained symptoms and a diagnosis of ALA compressing the inferior vena cava (IVC) with subsequent thrombus formation and pulmonary thromboembolism (PTE) was made. He was managed successfully with antibiotics, percutaneous aspiration and anticoagulation.

## Introduction

The primary site of infection by *Entamoeba histolytica* is the colonic mucosa and it is acquired by ingestion of cysts through contaminated food [1]. Amebic liver abscess (ALA) is the most common extra-intestinal manifestation, occurring in around 1% of patients due to migration of the amebic trophozoites to liver through the portal venous system. Simultaneous diarrhea is seen in only a quarter of the patients, although many patients have had diarrhea in the preceding few months. Majority of the patients have a single abscess and the most common location is posterior surface of the right lobe. It can rupture into the surrounding body cavities and, when large enough and located in close proximity, it can also cause compression of hepatic veins or inferior vena cava (IVC) [2]. In this report, we are describing ALA as an intriguing cause of pulmonary thromboembolism (PTE) secondary to IVC compression and thrombosis.

## Case presentation

A 26-year-old male presented with fever and upper abdominal pain for nine days. Two days prior to presentation, he had developed exertional chest pain and dyspnea. There was no history of diarrhea. He had no significant past medical, surgical and travel history, and there was no family history of thrombotic disorders. On presentation, he was febrile and had a tender abdomen. He had tachycardia with pulse rate of 110 per minute and blood pressure was 110/70 mm Hg. Pulse oximetry revealed capillary blood oxygen saturation of 94% while breathing room air.

**Figure 1:**
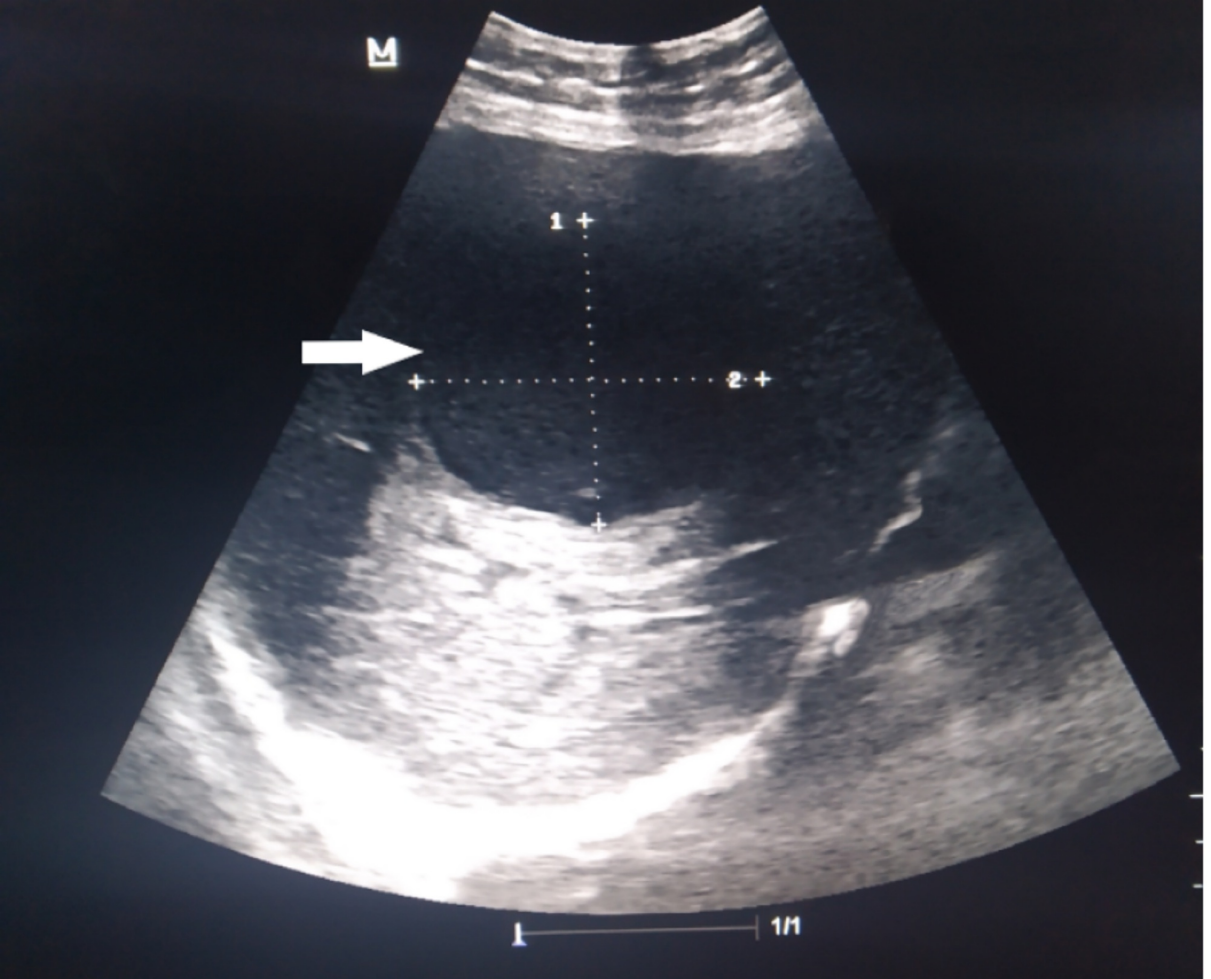
Ultrasound of the liver showing a hypodense lesion (arrow) in the right lobe.

**Figure 2:**
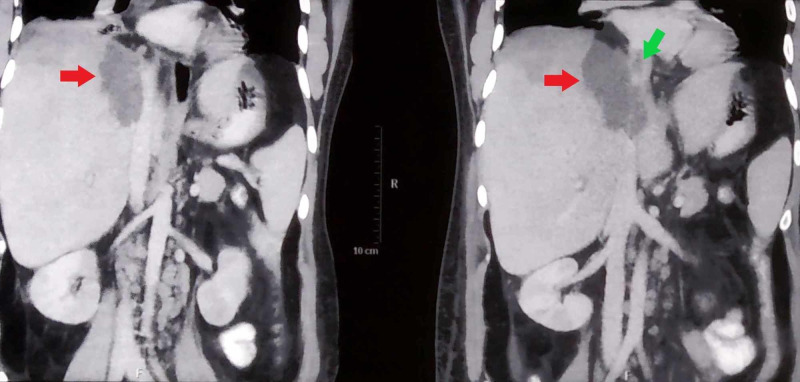
Coronal sections of computed tomographic (CT) scan of abdomen showing the liver abscess (red arrows) and thrombus in the adjacent inferior vena cava (green arrow).

**Figure 3:**
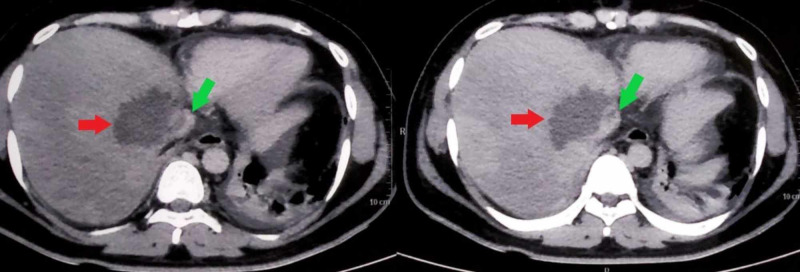
Axial sections of computed tomographic (CT) scan of abdomen showing the liver abscess (red arrows) and thrombus in the adjacent inferior vena cava (green arrows).

**Figure 4:**
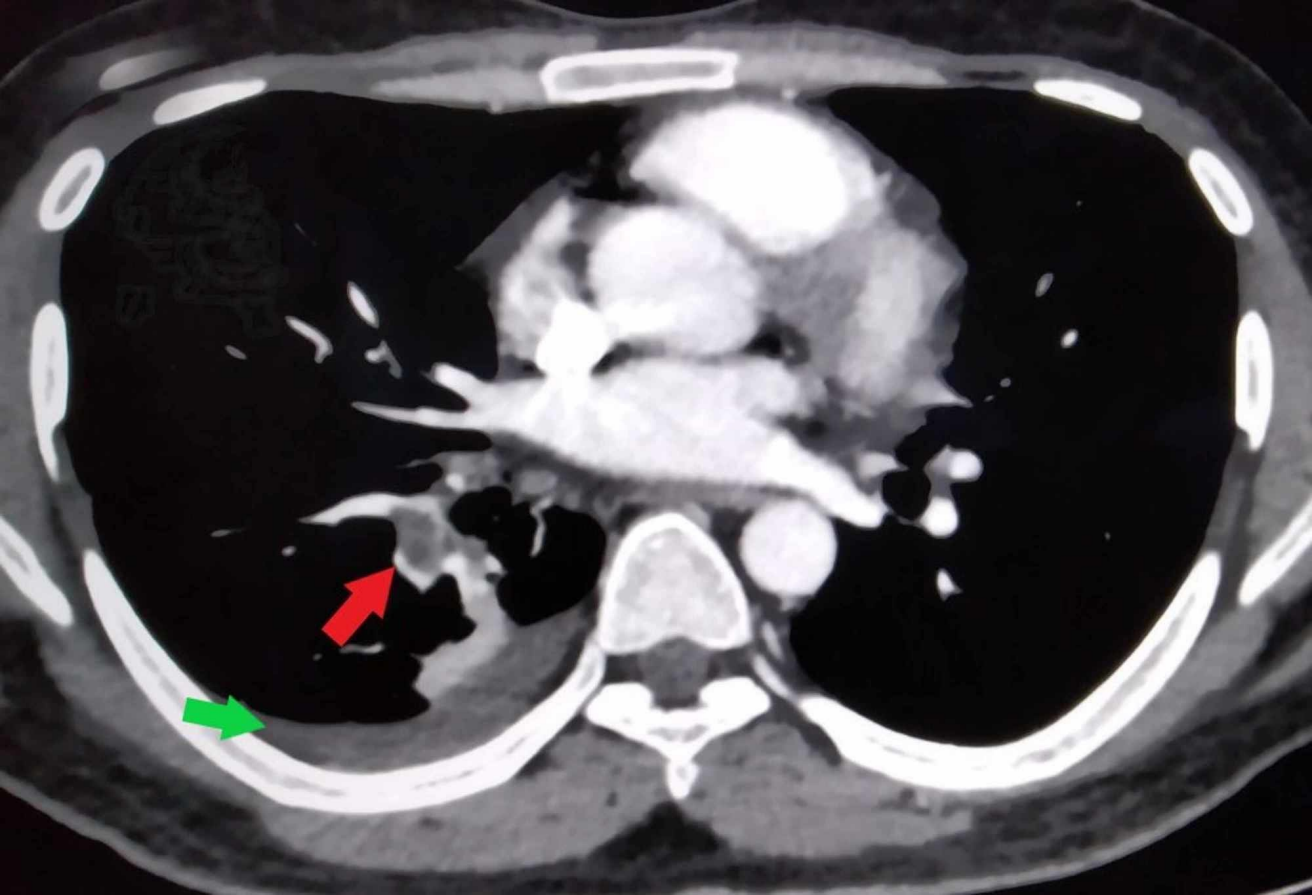
Axial section of computed tomographic (CT) angiogram of lungs showing pleural effusion (green arrow) and thrombus (red arrow) in a segmental branch of the right pulmonary artery.

His electrocardiogram was normal and chest radiograph revealed minimal pleural effusion on the right side. Blood investigations revealed an elevated white blood cell count of 17.5 x 10^9^/L (normal range: 4-12 x 10^9^/L) and mildly deranged liver functions with aspartate transaminase (AST) elevated to 69 U/L (normal range: 5-40 U/L), alanine transaminase (ALT) elevated to 71 U/L (normal range: 5-40 U/L) and alkaline phosphatase (ALP) elevated to 350 U/L (normal range: 30-140 U/L). On ultrasound scan of abdomen, the liver was found to be enlarged to 20 cm with a hypoechoic lesion in the right lobe (Figure 1). The serologic diagnosis of ALA was confirmed with indirect hemagglutination. Contrast-enhanced computed tomographic (CT) scan of abdomen showed hepatomegaly with an abscess of 8.6 cm x 3.8 cm in segment VIII of the right lobe causing IVC compression with a thrombus inside its lumen (Figures 2, 3). CT pulmonary angiogram revealed a filling defect in right lower lung segmental artery (Figure 4). Echocardiography and venous Doppler of the legs did not reveal any abnormality.

The treatment comprised of intravenous metronidazole (800 mg thrice a day) along with the oral luminal amebicide agent, diloxanide furoate (500 mg thrice a day) for 10 days. Percutaneous needle aspiration of the abscess was carried out. Anticoagulation with weight-based dose of subcutaneous enoxaparin (60 mg twice a day) was simultaneously started and later bridged to warfarin to maintain international normalized ratio (INR) of 2-3. The patient became asymptomatic and anticoagulation was discontinued after three months.

## Discussion

The cardinal clinical features of ALA are fever, pain in the right upper quadrant of abdomen, nausea and vomiting [3]. Some patients can present with a prolonged illness of several weeks with anorexia, weight loss and low-grade fever. The abscess can extend superiorly and lead to pleural effusion, empyema, pneumonitis or bronchopleural fistula. Rupture into peritoneum and pericardium can lead to peritonitis and cardiac tamponade, respectively. Compression of the biliary tree can lead to obstructive jaundice. IVC compression and thrombus formation is rare and only isolated case reports have been published, so the exact incidence is not known [4,5]. It occurs due to relative stasis of blood and spill-over of the inflammatory process into the vessel wall. The thrombus can propagate and extend superiorly into the heart or inferiorly into the iliac veins. This is in contrast to hepatic and portal vein thrombosis which are not infrequent [6].

McKenzie et al. have documented a case similar to one described in this report and the patient had bilateral multiple pulmonary emboli [7]. Extension of thrombus from the IVC into the right atrium is a potentially lethal complication. Prendki et al. have described a case with extensive IVC thrombosis which extended into the right atrium along with embolism into the right main pulmonary artery [8]. The patient was managed with anticoagulation alone. Gupta et al. and Khan and Ameen Rauf have described cases with right atrial thrombi secondary to ALA that required open surgical thrombectomy [9,10]. In the covert presentation of a case described by Martin et al., the patient had two small hepatic lesions (3.2 cm and 1.9 cm) along with thrombosis of the IVC, right accessory hepatic vein and bilateral iliac veins [11]. The lesions were initially felt to represent hepatocellular carcinoma but it could not be confirmed as multiple biopsies were non-conclusive. The diagnosis of ALA was made four months later when one of the lesions enlarged to 10.4 cm x 12 cm and its aspiration yielded the characteristic anchovy sauce colored pus. The diagnosis was confirmed by serology.

The symptoms of breathlessness, cough and chest pain can occur due to the other pulmonary complications of ALA [3]. PTE can clandestinely present with the same symptoms, so a high index of suspicion is necessary for diagnosis. In the case under consideration, minimal pleural effusion on chest radiograph along with IVC thrombosis prompted the search for an alternate pathology.

The medical management is done by metronidazole or tinidazole combined with a luminal amebicide to prevent relapse [1]. Percutaneous aspiration or pig-tail drainage is indicated when there is impending rupture, compression of biliary tree or great vessels, failure of antibiotic therapy or when the abscess is large. Although no fixed cut-off is recommended, abscesses greater than 8 cm in diameter usually require drainage. Open surgical intervention is rarely required in ALA [1]. Rupture into the pericardium is an emergency and immediate drainage is required to prevent tamponade. Intercostal drainage is indicated in case of rupture into the pleural space.

Standard anticoagulation protocol was followed in our case. Warfarin was discontinued after three months; however, the duration needs to be individualized and prolonged therapy may be needed for persistent symptoms or if an underlying hypercoagulable state is detected [8]. Massive PTE is also a possibility and that may necessitate thrombolytic therapy or mechanical thrombectomy.

## Conclusions

This report describes a young male with ALA who had dyspnea out of proportion to the finding of mild right-sided pleural effusion. Diagnosis of IVC thrombosis and PTE was made on CT scan. He was treated with percutaneous aspiration of the ALA, anti-amebic drugs and anticoagulation. The crux of this case is that PTE is a potential complication of ALA. Respiratory symptoms may be falsely attributed to pleural effusion, empyema or pneumonitis, and it is worthwhile to investigate for PTE early in the disease course when symptoms are not fully expounded, especially in the presence of IVC or hepatic vein thrombosis.
